# The Hypomorphic Variant p.(Gly624Asp) in *COL4A5* as a Possible Cause for an Unexpected Severe Phenotype in a Family With X-Linked Alport Syndrome

**DOI:** 10.3389/fped.2019.00485

**Published:** 2019-11-26

**Authors:** Eva Pauline Macheroux, Matthias C. Braunisch, Stephanie Pucci Pegler, Robin Satanovskij, Korbinian M. Riedhammer, Roman Günthner, Oliver Gross, Mato Nagel, Lutz Renders, Julia Hoefele

**Affiliations:** ^1^Institute of Human Genetics, School of Medicine, Technical University of Munich, Munich, Germany; ^2^Department of Nephrology, School of Medicine, Technical University of Munich, Munich, Germany; ^3^Clinic of Nephrology and Rheumatology, University Medical Center Goettingen, University of Goettingen, Goettingen, Germany; ^4^Center for Nephrology and Metabolic Medicine, Weisswasser, Germany

**Keywords:** Alport syndrome, *COL4A5*, p.Gly624Asp, ESRD, hearing impairment

## Abstract

**Background:** Alport syndrome (AS) is a progressive kidney disorder leading to end stage renal disease (ESRD). Extrarenal symptoms like hearing loss and ocular changes can be observed. Approximately 85% of the patients carry pathogenic variants in *COL4A5* (X-linked inheritance). The variant c.1871G>A, p.(Gly624Asp) in *COL4A5* is described in the literature as a hypomorphic variant associated with thin basement membrane nephropathy (TBMN). ESRD was only seen rarely at a median age of 50 years and extrarenal manifestations have only been described in single cases.

**Case report and Methods:** This is a report on a family with X-linked AS. In the female index patient, microscopic hematuria, and proteinuria were observed beginning at the age of 20 years and 41 years, respectively. Microscopic hematuria was also present in the daughter (from 6th month of life), the son (from 22nd month of life), the mother and the maternal grandniece. Proteinuria was observed in the maternal aunt and paternal grandmother. The father of the index patient, a paternal uncle and a second cousin presented with ESRD at the age of 49, 34, and 70 years of life, respectively. Extrarenal manifestations were absent in the whole family. In the index patient, her children and her mother molecular diagnostics were performed using Sanger and exome sequencing.

**Results:** In all examined family members the variant c.1871G>A, p.(Gly624Asp) in *COL4A5* was identified. With the exception of the index patient, who was homozygous for this variant, all family members carried the variant heterozygously, or hemizygously. A different or additional monogenic hereditary nephropathy could not be detected by exome sequencing of the index patient.

**Discussion:** This is the first report of a patient with the variant p.(Gly624Asp) in *COL4A5* in a homozygous state. The variant was previously reported as a mild variant requiring dialysis in less than 10%. The family presented, however, with a more severe clinical course. We therefore suggest to question the term “hypomorphic” in the context of the variant p.(Gly624Asp) although molecular diagnostics could not be done in all affected family members.

## Introduction

Alport syndrome (AS) is a rare hereditary kidney disorder with an estimated prevalence of 1:5,000 (X-linked form). It is characterized by hematuria, proteinuria, and progressive renal failure leading to chronic kidney disease (1–7). AS causes end stage renal disease in about 1–2% of patients in infancy, adolescence, and young adulthood. Frequent extrarenal manifestations are sensorineural hearing loss (60–80% of patients), ocular changes (lenticonus of the anterior lens capsule, retinopathy; 25–40% of patients) and rarely, mental retardation, and leiomyomatosis (8, 9).

AS is caused by pathogenic variants in the genes *COL4A3, COL4A4*, and *COL4A5*, encoding the α3, α4, and α5 chain of collagen type IV (2). In ~85% of patients with AS, pathogenic variants in *COL4A5* on chromosome Xq22.3 are causative (10, 11). AS can also be inherited in an autosomal recessive pattern (about 15% of the cases; biallelic pathogenic variants in *COL4A3* and *COL4A4*). Other presumed modes of inheritance are autosomal dominant or a digenic inheritance (12, 13).

Because of the common X-linked inheritance, mostly males are affected and show a distinct genotype-phenotype correlation (14). Missense or in-frame variants lead to a milder progress of disease with a later onset of end-stage renal disease (ESRD) (37 years) compared to splice-site and truncating variants which lead to a more severe phenotype with early onset of ESRD (25–28 years). Additionally, a strong relationship between variant position and age at ESRD has been demonstrated, with younger age at ESRD associated with pathogenic variants at the 5′ end of the gene. Furthermore, patients with splice-site variants and truncating variants had two-fold greater odds of developing ocular changes and/or hearing impairment than those with missense variants (14, 15).

Some variants in the *COL4A3-5* genes are described as hypomorphic as they are not fully destructive and therefore obtain a residual protein function. One of these variants is p.(Gly624Asp) in *COL4A5* which has been described in the context of thin basement membrane nephropathy (TBMN) in males. The clinical phenotype was comparable to a heterozygous carrier of a variant in *COL4A3* or *COL4A4* (16, 17).

This report describes a family with several individuals with symptoms ranging from proteinuria up to ESRD requiring dialysis. In some of the family members the variant p.(Gly624Asp) in *COL4A5* could be confirmed. Furthermore, this is the first description of a female patient with this hypomorphic variant in a homozygous state. Additionally, this is the first report of a female patient with a homozygous variant in *COL4A5* at all.

## Materials and Methods

### Clinical Case Information

The study was approved by the local Ethics Committee of the Technical University of Munich and performed according to the standards of the 2013 Helsinki Declaration. Written informed consent was obtained from the index patient and their relatives for publication. Clinical and phenotype information was gathered out of clinical reports and medical history.

### Genetics

**Figure 1:**
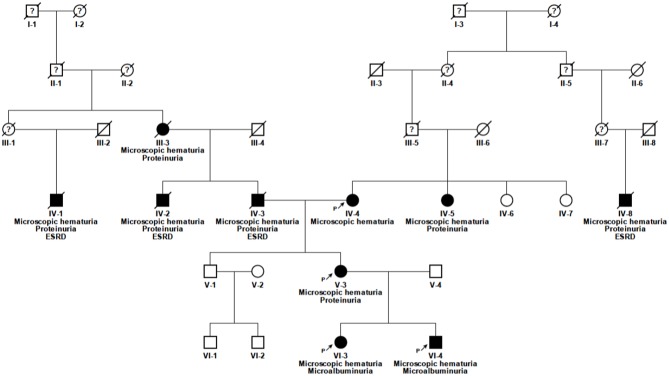
Pedigree of the family. Solid symbols, affected individuals; circles, females; squares, males; n.d., no data available; P, proband; ?, unknown medical history.

Blood samples were collected after written informed consent. Genomic DNA was extracted from peripheral blood of the index patient (Figure 1, V-3) and her children (Figure 1, VI-3 and VI-4) using the Gentra Puregene Blood Kit (Qiagen, Hilden, Germany) according to manufacturer's instructions.

#### Sanger Sequencing

In the index patient exon 1–51 of *COL4A5* were examined followed by exon 1–52 of *COL4A3* and exon 1–48 of *COL4A4* using direct DNA sequencing on both strands applying the dideoxy chain termination method on an ABI capillary sequencer 3730 (Applied Biosystems, Foster City, USA). Primers were designed by Primer3 program (http://frodo.wi.mit.edu/primer3/input.htm). For segregation analysis, subsequent targeted sequencing was performed in mother and children of the index patient in exon 25 of the *COL4A5* gene. DNA alignment and sequence variant analysis were carried out using the Sequence Pilot^CE^ software (JSI Medical Systems GmbH, Kippenheim, Germany) and compared to EMBL (European Molecular Biology Laboratory) and GenBank databases as well as our in-house database. All variants were validated in a second independent DNA sample.

#### Exome Sequencing

Exome sequencing was additionally performed in the index patient to examine pathogenic variants and genetic modifier in other genes responsible for the renal phenotype and its severity using a Sure Select Human All Exon 60Mb V6 Kit (Agilent) and a HiSeq4000 (Illumina) as previously described (18, 19). Reads were aligned to the UCSC human reference assembly (hg19) with BWA v.0.5.8. More than 98% of the exome was covered at least 20x. Single-nucleotide variants (SNVs) and small insertions and deletions were detected with SAMtools v.0.1.7. For the analysis of autosomal dominant and mitochondrial variants, only variants with a minor allele frequency (MAF) of less than 0.1% in the in-house database of the Helmholtz center Munich containing over 16,000 exomes were considered. For the analysis of autosomal recessive and X-linked variants (homozygous, hemizygous or putative compound heterozygous), variants with a MAF of <1.0% were considered. As there are pathogenic alleles in hereditary nephropathies with a MAF of more than 1.0% like the *NPHS2* p.Arg229Gln or the *NEPH3* p.Val353Met allele (20–22), an additional search for recessive and X-linked variants with a MAF up to 3% was used. Variants were classified in accordance to the classification of the American College of Human Genetics (AMCG) (23, 24). Exome depth was used as a CNV calling algorithm (25).

## Results

### Clinical Phenotype

#### Index Patient

The 52 year old female index patient is of Caucasian origin (Figure 1, V-3). She is of non-consanguineous German ancestry and presented to our department with microscopic hematuria and proteinuria. The further clinical presentation was unremarkable, besides a nasal septum operation with a tonsillectomy at the age of 10 years, varicose veins at the right lower leg and an atrial septal aneurysm with patent foramen ovale. Notably, there was no report of any extrarenal involvement. At the age of 20 years the index patient was first diagnosed with microhematuria, followed by microalbuminuria (132 mg/24 h) at the age of 34 years and by proteinuria (822 mg/g creatinine) first seen at the age of 41 years. An ultrasound examination performed at the age of 34 years showed morphologically normal kidneys. Therapy with angiotensin-converting enzyme (ACE) inhibitor was initiated at the age of 39 years which was switched to an AT1 antagonist because of ACE inhibitor-induced cough.

#### Relatives

The daughter of the index patient (VI-3) first presented with microhematuria at the age of 6 months and microalbuminuria (182 mg/g creatinine) at the age of 4 ½ years (see Table 1). She is currently 22 years of age and without medication. The son of the index patient (VI-4) showed microscopic hematuria and microalbuminuria (134 mg/g creatinine) since the age of 22 months. He is currently 20 years of age and is treated with AT1 antagonist since the age of 19 years (microalbuminuria <300 mg/g creatinine). The father of the index patient (IV-3) was on dialysis since the age of 49 years, as well as a paternal uncle (IV-2) and a paternal grand cousin (IV-1) (34 and 70 years of age, respectively). The father of the index patient died from stroke which occurred after kidney transplantation. The mother of the index patient (IV-4) is known to have microscopic hematuria and an eGFR of 65 ml/min/1.73 m^2^ at the current age of 78 years. The treating physician denied to perform urine analysis in this relative so far. The paternal grandmother (III-3) was known having proteinuria. The maternal aunt (IV-5) developed proteinuria after giving birth to twins at the age of 23 years. Extrarenal manifestations could not be detected in any of the family member.

**Table 1:** Overview about the p.(Gly624Asp) in *COL4A5*-associated clinical phenotype and the genetic zygosity of the affected family members.

**Individual**	**Zygosity**	**Current age or age at death**	**Age at microscopic hematuria**	**Age at mircoalbuminuria or proteinuria**	**Age at dialysis**	**Age at transplantation**
III-3	n.d.	89 years; *^†^*	n.d.	n.d.	n.d.	n.d.
IV-1	n.d.	75 years; *^†^*	n.d.	n.d.	70 years	n.p.
IV-2	n.d.	34 years; *^†^*	n.d.	n.d.	33 years	n.d.
IV-3	n.d.	56 years; *^†^*	n.d.	n.d.	49 years	56 years
IV-4	heterozygous	78 years	n.d.	n.d.	n.y.	n.y.
IV-4	n.d.	84 years	n.d.	23 years	n.y.	n.y.
IV-8	n.d.	n.d.; *^†^*	n.d.	n.d.	n.d.	n.d.
IV-3	homozygous	52 years	20 years	41 years	n.y.	n.y.
VI-3	heterozygous	21 years	6 months	4 years	n.y.	n.y.
VI-4	hemizygous	19 years	1 year 10 months	1 year 10 months	n.y.	n.y.

n.d., no data available; n.p., not performed; n.y., not yet;

† *, deceased*.

### Genetic Analysis

Pathogenic variants in *COL4A3* and *COL4A4* could not be identified. Sanger sequencing of *COL4A5* (NM_033380.2) in the index patient revealed the homozygous missense variant c.1871G>A, p.(Gly624Asp) (Figure 1, V-3). The variant is located in exon 25. The variant leads to a change of an evolutionarily highly conserved nucleotide at the second position of codon 624. The son and the daughter of the index patient carry the same variant in a hemizygous and a heterozygous state, respectively (Figure 1, VI-3 and VI-4). This variant was also present in the mother of the index patient (Figure 1, IV-4). Exome sequencing of the index patient did not reveal any other pathogenic variant responsible for the renal phenotype in this family. Modifier variants in other podocyte genes like *NPHS2* or *NEPH3* were also not identified.

## Discussion

In all available affected family members the already known variant p.(Gly624Asp) in *COL4A5* most likely causative for their renal phenotype was identified. The presence of a glycine residue as every third amino acid in the collagenous domain of COL4A3/4/5 is essential. Therefore, most of the pathogenic variants identified in patients with AS affect a glycine residue. However, the variant p.(Gly624Asp) is described in the literature as a hypomorphic variant leading to the mild clinical phenotype of TBMN in males and females caused by a residual function of the remaining protein. In the patients carrying this variant ESRD is very rare and only seen in single patients at a median age of 50 years (16, 17). The variant is also reported in the genome Aggregation Database (gnomAD) with an overall allele frequency of 1 in 11,438 individuals. In Europeans (non-Finnish), the allele frequency is 1 in 5105, including four hemizygotes and no homozygotes. In this family all affected and molecular confirmed individuals developed microalbuminuria or proteinuria later in life. Additionally, all affected adult male individuals presumably carrying this variant presented with ESRD. In contrast to the available literature, this observation suggests that this variant is not as mild as assumed.

As variants in other genes can mimic a similar clinical and histological phenotype of AS and albeit the pedigree seems compatible with an X-linked inheritance pattern, a second monogenic disease was not identified by exome sequencing. And even if exome sequencing cannot absolutely exclude other pathogenic variants and not in all affected relatives molecular analysis could be performed, another genetic cause seems unlikely in this family.

Extrarenal manifestations are a common feature in AS patients observed in 25% (ocular changes) and 80% (sensorineural hearing loss) of the cases, respectively. In the literature only two patients carrying the hemizygous variant p.(Gly624Asp) developed late onset hearing impairment so far and one patient ocular lesions (16). As the affected individuals in our family did also not develop any extrarenal malformations, this observation can support the rare occurrence of extrarenal manifestations.

To our knowledge, this is the first report on a patient carrying the variant p.(Gly624Asp) in homozygous state and with a homozygous variant in *COL4A5* at all. Female patients with a heterozygous variant in *COL4A5* (X-linked AS) have been traditionally described as carrier female subjects even though with variable intra- and interfamilial penetrance, showing a broad spectrum of clinical symptoms ranging from mild microscopic hematuria to severe AS (26–29). In contrast to male patients, genotype-phenotype correlation is less-well described in females so far but loss-of-function variants appear to result in a more severe phenotype (27, 30). As possible causes for phenotypic variability in X-linked AS, the type of variant, degree of mosaicism following lyonization of the X chromosome, and genetic modifiers are discussed, among other things (13, 26, 31, 32).

As the female index patient of the reported family is genetically comparable with a male patient, and even if other genetic causes (e.g., digenic inheritance, intronic variants, genetic modifiers) cannot be excluded for sure in her affected relatives and non-genetic factors might be different, she might develop ESRD according to her affected male relatives.

As this variant seems to lead to a different clinical phenotype and some of the carriers seem to have a more severe phenotype than known in the literature so far, a registry study solely collecting patients with the variant p.(Gly624Asp) should be initiated to answer the open question concerning the severity of this variant. Genetic modifiers in other genes like *NPHS2* or *NEPH3* could be one possible cause for an aggravation of the symptoms but were not seen in the present case (20–22). However, according to this report the term “hypomorphic” in the context of this variant should be questioned.

## Conclusion

This report presents a severely affected family with the so far called “hypomorphic” variant p.(Gly624Asp) in *COL4A5*. It also underlines that patients with a variant classified as hypomorphic can present with AS and therefore have to be monitored closely as well as treated accordingly.

## Data Availability Statement

The datasets analyzed in this manuscript are not publicly available. Requests to access the datasets should be directed to JH, julia.hoefele@tum.de.

## Ethics Statement

The studies involving human participants were reviewed and approved by Ethics Committee, Technical University of Munich, Munich, Germany. Written informed consent to participate in this study was provided by the participants' legal guardian/next of kin. Written informed consent was obtained from the individual(s), and minor(s)' legal guardian/next of kin, for the publication of any potentially identifiable images or data included in this article.

## Author Contributions

MB, KR, and JH analyzed and interpreted the patient data regarding the genetic and clinical findings. EM, SP, RG, and JH wrote the manuscript. KR, RS, and MN performed the molecular diagnostics. OG conducted in-patient treatment. EM, MB, LR, KR, and RG contributed important intellectual content during manuscript drafting and revision. All authors accept accountability for the overall work by ensuring that questions pertaining to the accuracy or integrity of any portion of the work are appropriately investigated and resolved. Text revision was performed by all authors. All authors read and approved the final manuscript.

### Conflict of Interest

The authors declare that the research was conducted in the absence of any commercial or financial relationships that could be construed as a potential conflict of interest.

## Abbreviations

ACE: angiotensin-converting enzyme
AS: Alport syndrome
CNV: copy number variant
DNA: deoxyribonucleic acid
ESRD: end-stage renal disease
gnomAD: genome Aggregation Database
SNP: single nucleotide polymorphism
TBMN: thin basement membrane nephropathy.
